# Effectiveness of locally produced ready-to-use supplementary foods on the prevention of stunting in children aged 6–23 months: a community-based trial from Pakistan

**DOI:** 10.1017/S0007114523002702

**Published:** 2024-04-14

**Authors:** Sheraz Fazid, Zia Ul Haq, Basharat Hussain Gillani, Abdul Jalil Khan, Muhammad Naseem Khan, Aslam Khan, Cecilia Garzon, Ijaz Habib, Mahamadou Tanimoune, Yasir Ihtesham, Adrian H. Heald

**Affiliations:** 1 Institute of Public Health & Social Sciences, Khyber Medical University, Peshawar, Pakistan; 2 Institute of Health & Wellbeing, University of Glasgow, Glasgow, UK; 3 Department of Popualtion Medicine, College of Medicine, Qatar University, Doha, Qatar; 4 Institute of Basic Medical Sciences, Khyber Medical University, Peshawar, Pakistan; 5 World Food Program, Pakistan; 6 Department of Diabetes and Endocrinology, Salford Royal Hospital, Salford, UK; 7 The School of Medicine, Manchester Academic Health Sciences Centre, University of Manchester, Manchester, UK

**Keywords:** Children, Community, Length-for-age Z score, Malnutrition, Pakistan, Stunting

## Abstract

Undernutrition is a major public health problem in developing countries. Around 40·2 % of children are stunted in Pakistan. This longitudinal study aimed to assess the effectiveness of locally produced ready-to-use supplementary foods in the prevention of stunting by detecting change in of children in intervention *v*. control arm against the 2006 WHO growth reference. A community-based non-randomised cluster-controlled trial was conducted from January 2018 to December 2020 in the district of Kurram, Khyber Pakhtunkhwa, Pakistan. A total of 80 clusters (each cluster comprising ≈ 250–300 households) were defined in the catchment population of twelve health facilities. Children aged 6–18 months were recruited *n* 1680. The intervention included a daily ration of 50 g – locally produced ready-to-use-supplementary food (Wawa-Mum). The main outcome of this study was a change in length for age z-score (LAZ) *v*. WHO growth standards. Comparison between the interventions was by *t* test and ANOVA. Cox proportional hazard models were used to assess the association between stunting occurrence and the utilisation of locally produced supplement. Out of the total 1680, fifty-one out of the total 1680, 51·1 out of the total 1680 and 51·1 % (*n* 859) were male. Mean age 13·9 months (sd + 859) were male. Mean age 13·9 months (sd + –4·4). At baseline, 36·9 % (*n* 618) were stunted. In the intervention group, mean LAZ score significantly increased from −1·13(2·2 sd) at baseline to −0·93(1·8 sd) at 6-month follow-up (*P* value 0·01) compared with the control group. The incidence rate of stunting in the intervention arm was 1·3 *v*. 3·4 per person year in the control arm. The control group had a significantly increased likelihood of stunting (Hazard Ratio (HR) 1·7, 95 % CI 1·46, 2·05, *P* value < 0·001) *v*. the intervention group. Locally produced ready-to-use supplementary food is an effective intervention for reducing stunting in children below 2 years of age. This can be provided as part of a malnutrition prevention package to overcome the alarming rates of stunting in Pakistan.

Undernutrition is one of the major public health problems that affect children, especially those below 5 years of age, mainly in developing countries. Malnutrition along with poverty and disease acts in a vicious cycle. These factors are linked to each other in such a way that each of these factors contributes to the presence of others. Malnutrition occurs as a result of a deficiency of one or more nutrients including iodine, protein or vitamin A. Inadequate energy intake leads to stunting and wasting^(1)^.

Stunting or low height/length for age is caused by chronic malnutrition^(2)^. It is mainly an irreversible outcome of inadequate nutrition for a prolonged period and of repeated episodes of infection, specifically during the first 2 years of a child’s life. The long-term effects of stunting on individuals and societies lead to irreversible outcomes including poor cognitive and physical growth of the child followed by decreased productivity and an increased risk of non-communicable and comorbid diseases^(3)^. There is likely also limited access to full time education, compromised examination performance, together with an increased likelihood of living a life in poverty^(4)^.

Globally, 149·2 million children below the age of 5 years are suffering from chronic malnutrition (UNICEF 2022). Chronic malnutrition along with inequality and poverty passes from one generation to another. It is further worsened by illness, resulting in slowing down of metabolism and ultimately deleterious effects on the child’s immune system^(5)^.

The National Nutrition Survey of Pakistan (2018) revealed that 40 % (12 million) of children below 5 years of age are stunted with the prevalence is little higher in males (40·9 %). The burden of chronic malnutrition in below 2-year children of Khyber Pakhtunkhwa province is 40 %, and the newly merged districts (previously known as the federally administered tribal areas) represent the worst situation across the country, with almost every other child under 5 years of age stunted (48·3 %)^(6)^.

Child growth is markedly affected by socio-demographic factors. Throughout the world, different patterns of malnutrition (undernutrition) are observed with the highest prevalence of acute and chronic malnutrition in Asia. Such patterns of malnutrition across the world reveal that there are multiple causes associated with malnutrition and in the developing world such constraints are further exacerbated by natural and man-made disasters^(7)^.

Very little consideration is given to the issue of decreased linear growth in comparison to international recommended standards (stunting) in humanitarian and protracted emergency contexts with the focus of policy, research and programming directed towards the treatment of acute malnutrition in children^(8)^.

In the developing world, studies on the efficacy of locally produced food-based interventions for the prevention of chronic malnutrition exist, but they are often carried out in controlled conditions/situations, and the generalisability of those results cannot measure the impact of such interventions under ‘field’ conditions. Control and prevention of chronic malnutrition in Pakistan is the top national priority due to its direct relationship with human capital (NNS-2018). In this context, we conducted this community-based trial to assess the effectiveness of locally produced supplementary food on the prevention of chronic malnutrition in children below 2 years of age.

## Methods

### Study design, settings and ethics

A community-based non-randomised cluster-controlled trial was conducted in the district of Kurram, Khyber Pakhtunkhwa Province, Pakistan, from January 2018 to December 2020. This study was conducted according to the guidelines laid down in the Declaration of Helsinki, and all procedures involving human subjects/patients were approved by the ethical review board of the Khyber Medical Unviersity Ref No: DIR.KMU-EB/SP/000427. Written informed consent was obtained from all subjects. This trial is registered with International Standard Randomised Control Trial Number recognised by World Health Organization International Clinical Trial Registry Platform and International Committee of Medical Journal Editors under the registration number: ISRCTN94319790^(9)^.

### Intervention

Wawa Mum was provided as supplementary food to the study participants in the intervention arm. The active ingredients include roasted chickpeas, vegetable oil (palm, rapeseed), dry skimmed milk, sugar, vitamins and minerals, emulsifier and antioxidants. It is a locally produced lipid-based nutrition supplement (LNS) given on daily basis as part of general food distribution. Each sachet weighs 50 g covering about ¼ of daily energy requirements of children 6–23 months of age. It also comprises most of the micronutrients according to recommended daily allowance^(10)^. It provides a minimum of 255 kcal of energy and most micronutrients which are essential for the child growth and development according to the recommended daily allowance. It has high nutrient content than other fortified blended food and have no limitations in terms of texture, size and viscous nature. It is also considered comparatively more result prone supplement in this context^(11)^ (online Supplementary Table 1).

Health messages by the Department of Health Khyber Pakhtunkhwa for care givers of children below 5 years of age were delivered throughout the study to both intervention and control groups. These messages focused on improving child feeding practices (infant and young child feeding practices), immunisation of the child and water sanitation and hygiene practices.

### Outcomes

The main outcome of this study was a change in length for age z-score (LAZ) of the study participants against the WHO-2006 growth standards. Anthropometric data were collected through a trained data collection team using a height board (From UN World Food Program-Pakistan) to the nearest 0·1 cm, weight measurements using Seca digital weighing scales to the nearest 0·1 kg and mid upper arm circumference using flexible insertion tape (from WFP-Pak). Each measurement was recorded three times and then the average was taken.

### Cluster identification

A total of 122 clusters were formed in the catchment population of twelve health facilities using the Expanded Program on Immunisation Microplans for the Polio Eradication Initiative of the WHO. These microplans have detailed information on the number of households and a map. Each cluster comprised of 100–150 households. Intervention and control clusters were selected based on natural segregation which reduced the chances of contamination between intervention and control group.^(12)^

### Sample size

The sample size was calculated using the University of Aberdeen sample size calculator^(13)^ for cluster randomised trials by assuming an intra-cluster correlation coefficient of 0·06, a cluster size of twenty-three children (6–23 months) and eighty clusters to give 90 % power to detect a difference of 0·25 sd in LAZ score at a significance level of 0·05. The total estimated sample size was 1840 (920 in intervention arm and 920 in control arm).

### Eligibility criteria, recruitment, enrollment and consent

**Fig. 1.**
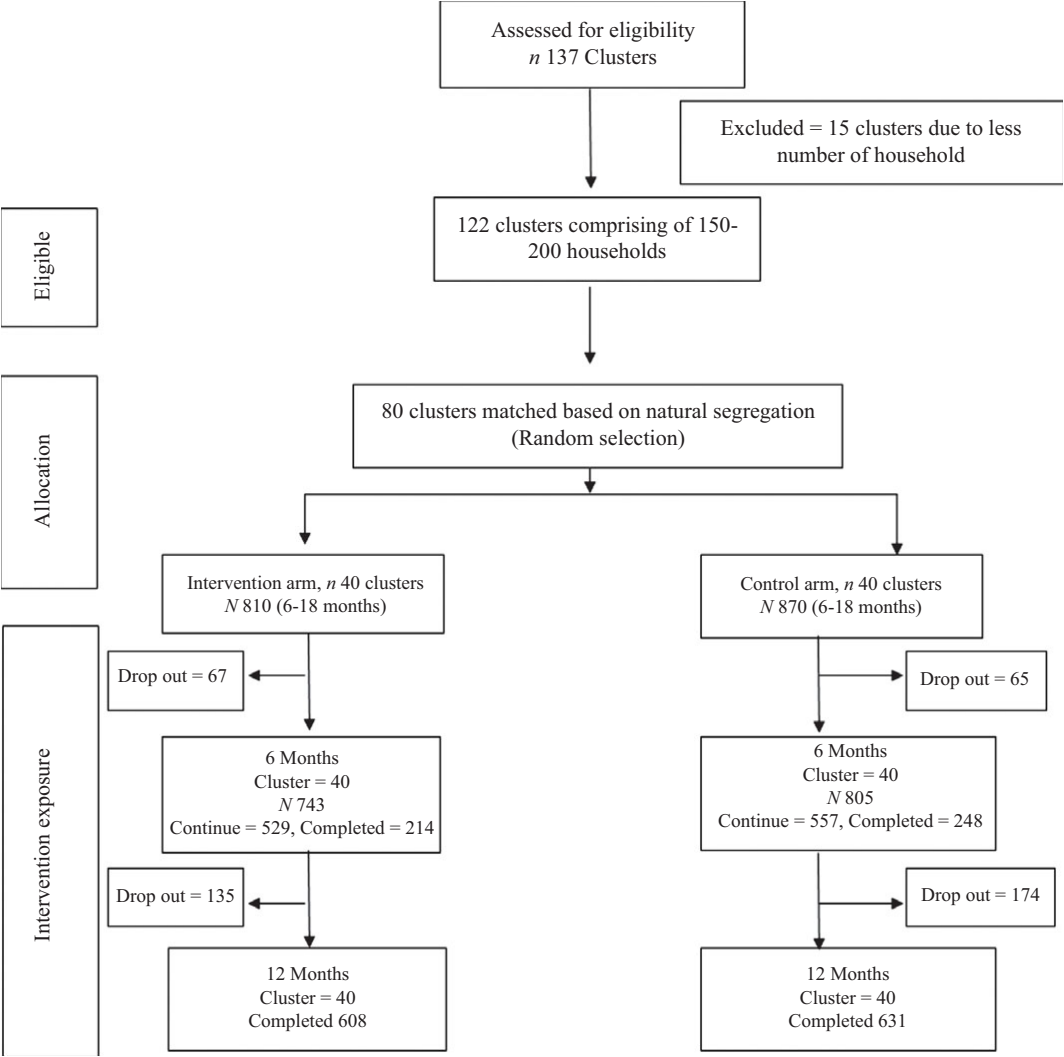
Consort diagram.

Children 6–18 months of age were recruited from these clusters using a multi-staged cluster sampling technique. A total of 122 clusters were formed in the study area. Among these clusters, eighty clusters were randomly selected, i.e. forty each in the intervention and control arms of the study. Then households were line listed in each of the identified cluster. Households having at least one child 6–18 months of age were selected using systematic random sampling. Children with congenital malformations and severe acute malnutrition were excluded from the study (Fig. 1).

### Data collection

Baseline data collection included demographics; household characteristics, socio-economic status, health services utilisation, infant and young child feeding, routine immunisation, morbidities, water sanitation and hygiene practices, household food insecurity and anthropometric assessment of the children. All the data including baseline and monthly follow-up data were collected at the community level by a trained data collection team with the support of lady health workers in the respective areas. In the intervention group, participants were provided with LNS and health messages, while in control group only health messages were given on monthly basis. Every month the caregivers of the children were reminded about giving the LNS to their children as part of regular food. All the study participants were followed for a period of 1 year after the implementation of intervention.

### Statistical analysis

All data were entered and analysed in STATA-15. Background characteristics at baseline were compared between the study arms using independent sample *t* test (for continuous variables) and *χ* ^2^ tests (for categorical variables).

Repeated measures ANOVA was used to detect change in LAZ at baseline, 6-month follow-up and endline (12 months) while satisfying the normality assumptions. *χ* ^2^ test was used to assess the association between chronic malnutrition and LNS (study arm).

Cox proportional hazard models were used to assess the association between stunting occurrence and the utilisation of LNS. The model was run in two stages: Unadjusted model and adjusted model (for potential confounders; baseline values of LAZ scores, GE of the child at enrollment, gender of the child, cluster identity, routine immunisation and socio-economic status).

## Results

A total of 1680 children were recruited out of the total 1860 screened participants. Among the excluded children (*n* 179), 48 were severe acute malnutrition (mid upper arm circumference < 11 cm) and 131 parents did not give consent to participate in the study. Children excluded due to severe acute malnutrition were referred to the stabilisation center at the District Headquarter hospital, Kurram. Out of the total 1680 recruited children, 810 and 870 were in the intervention and control arms, respectively.

Loss to follow-up after first follow-up (6 months) was 8·3 % and 7·5 % in the intervention and control arms which increased to 24·9 % and 27·5 % after end of follow-up (12 months), respectively.

### Baseline characteristics

Out of the total 1680 children, 81·14 % (*n* 1364) were living in joint families, 70·91 % (*n* 1192) mothers were illiterate, and majority were housewives 97·8 % (*n* 1642). On the socio-economic status assessment, 9·58 % (*n* 161) were living below poverty line and 44·74 % (*n* 752) were in the marginal group.

Mean age of the children at enrolment was 13·9 months (sd 4·4) and 51·13 % (*n* 859) were male. Mean height of the children was 73·02 cm (sd 7·68), and mean weight was 9·03 kg (sd 1·72). There was no significant difference between age, gender, height and weight of the children in the control and intervention arm at the time of enrollment (*P* value 0·37, 0·42, 0·05, 0·41, respectively).

The overall prevalence of stunting was 36·9 % (*n* 618) and wasting was 9·1 % (*n* 153). Among the intervention arm, the prevalence of stunting was 31·8 % (*n* 256) and of wasting was 8·9 % (*n* 72), whereas in the control group, prevalence of stunting was 41·7 % (*n* 362) and wasting was 9·2 % (*n* 80) (*P* value < 0·05). Children with complete routine immunisation constituted 56·24 % (*n* 942), and there was no difference between children in the intervention and control groups (*P* value 0·12). A total of 20 % (*n* 336) children had diarrhoea in the last 1 month. Children with diarrhoea in the last 1 month at enrollment comprised 17·7 % (*n* 143) in the intervention group and 22·18 % (*n* 193) in the control arm (*P* value 0·02) (Table 1).

**Table 1.** Baseline characteristics of the study participants (*n* 1680)

	Control *n* 870 (40 clusters)		Intervention *n* 810 (40 clusters)		Total *n* 1680 (80 clusters)		*P* value
	*n*	%	*n*	%	*n*	%	
Age, months							
Mean	13·9		13·8		13·9		0·37
sd	4·0		4·7		4·4		
Age groups							
Below 1 year	431	49·54	447	55·19	878	52·26	0·02
Above 1 year	439	50·46	363	44·81	802	47·74	
Gender							
Male	452	51·95	407	50·25	859	51·13	0·42
Female	418	48·05	403	49·75	821	48·87	
Weight in kg							
Mean	9·04		9·01		9·03		0·41
sd	1·78		1·65		1·72		
Length (cm)							
Mean	72·67		73·39		73·02		0·05
sd	7·93		7·38		7·68		
MUAC (cm)	13·29	1·27	13·67	1·13	13·47	1·23	0·10
LAZ score	–1·57	2·69	–1·13	2·18	–1·36	2·47	0·003
WLZ score	0·34	1·41	0·05	1·31	0·21	1·37	< 0·001
Stunting	362	41·7	256	31·8	618	36·9	< 0·001
Wasting	80	9·2	72	8·9	153	9·1	0·84
Complete immunisation	505	58·1	437	54·3	942	56·2	0·12
Diarrhoea in the last month	193	22·2	143	17·7	336	20·0	0·02
Socio-economic status							
Poor	506	58·16	407	50·25	913	54·35	0·001
Non-poor	364	41·84	403	49·75	767	45·65	

### Anthropometric outcomes

Table 2 shows the anthropometric outcomes of the study over 1-year period. It shows that the height of the participants in the intervention arm significantly changed from baseline to end of follow-up. The mean height increased from baseline 73·4 cm (6·2 sd) to 82·1 cm (5·7 sd) at sixth month and the difference 8·52 cm (8·2) was statistically significant (*P* value < 0·001). There was significant increase of 4·76 cm in the next 6 months (*P* value < 0·001). Among the control arm, there was mean increase of 7·58 cm (9·7 sd) at six-month follow-up and 5·18 (10·7 sd) at 12-month follow-up (*P* value < 0·001).

**Table 2.** Impact on child anthropometric outcomes over the study period (*n* 1680)

	0 months		6 months		12 months		*P* value
	Mean	sd	Mean	sd	Mean	sd	
Length in cm							
Control	72·74	6·98	81·12	7·36	85·71	6·60	< 0·001
Intervention	73·53	6·24	82·02	5·71	86·89	5·53	< 0·001
Overall	73·12	6·64	81·55	6·62	86·82	6·13	< 0·001
LAZ Score*							
Control	–1·52	2·21	–1·33	2·24	–1·45	1·80	0·1
Intervention	–1·18	1·87	–0·98	1·66	–1·08	1·44	0·03
Overall	–1·36	2·06	–1·16	1·99	–1·27	1·64	0·003

* LAZ, length for age z score.

In the intervention arm, mean LAZ score increased from −1·13 (2·2 sd) at baseline to −0·93 (1·8 sd) at 6-month follow-up and the difference was 0·19 (2·0 sd) (*P* value 0·01). In the control arm, there was no statistically significant improvement in the LAZ score at 6-month and 12-month follow-up (*P* value 0·18 and 0·09, respectively).

The incidence rate of stunting in the intervention arm was 1·3 per person year, and it was 3·4 per person year in the control arm.

Participants in the control arm (HR 1·7, *P* value < 0·001) were significantly more likely to experience stunting in the unadjusted model (Table 3, model 1). When adjusted for the child characteristics at the baseline, the association was statistically significant for male children (HR 1·3, *P* value 0·003), being stunted at baseline (HR 0·9, *P* value < 0·001) and participants in the control arm (HR 2·6, *P* value < 0·001) (Table 3, model 2).

**Table 3.** Cox proportional hazard models of the association between stunting overtime and child characteristics (*n* 1121)

	Model 1*			Model 2†			Model 3‡		
	HR	95 % CI	*P* value	HR	95 % CI	*P* value	HR	95 % CI	*P* value
Study arm									
Intervention	Reference			Reference			Reference		
Control arm	1·7	1·5, 2·1	< 0·001	2·6	1·8, 3·8	< 0·001	2·7	1·9, 3·9	< 0·001
Age in months	1·0	0·9, 1·1	0·08	1·0	0·9, 1·0	0·89	1·0	1·0–1·1	0·02
Gender of the child									
Female	Reference			Reference			Reference		
Male children	1·2	0·9, 1·4	0·08	1·3	1·1, 1·6	0·003	1·1	0·9, 1·4	0·16
LAZ score at baseline	0·8	0·8, 0·9	< 0·001	0·9	0·8–0·9	< 0·001	–		–
Immunisation at baseline									
Complete	Reference			Reference			Reference		
Incomplete	1·0	0·8, 1·3	0·95	1·1	0·8, 1·4	0·66	0·9	0·7, 1·2	0·76
Socio-economic status									
Non-poor	Reference			Reference			Reference		
Poor	1·1	0·9, 1·3	0·27	1·1	0·8, 1·3	0·79	1·1	0·9, 1·4	0·21
Cluster	1·0	1·00, 1·01	< 0·001	0·9	0·8, 0·9	0·002	1·0	0·9, 0·1	0·003
Breast-feeding at the time of enrollment									
No	Reference			Reference			Reference		
Yes	1·1	0·9, 1·3	0·21	1·1	0·9, 1·4	0·28	1·3	1·0, 1·6	0·02
Exclusive breast-feeding done									
No	Reference			Reference			Reference		
Yes	0·9	0·8, 1·2	0·88	0·98	0·8, 1·2	0·90	1·0	0·8, 1·2	0·93

LAZ, length for age z score.

* Model 1: Unadjusted model.

† Model 2: This model is adjusted for age of the child, gender, stunting at baseline, immunisation, socio-economic status, cluster ID and study arm.

‡ Model 3: This model is adjusted for age of the child, gender, immunisation, socio-economic status, cluster ID and study arm.

## Discussion

The locally produced ready-to-use supplementary food significantly improved the LAZ score of the children aged 6–23 months who received it, and the likelihood of stunting was significantly reduced over the course of the study. It also slowed down the child’s growth flattening by decreasing the incidence rate of stunting in the intervention arm. Overall, the stunting was reduced by 7 percent in the intervention arm in one-year period. Male children and those in the control arm were more likely to experience stunting. Along with this, the incidence of stunting after 12 months of follow-up was more than double in the children who did not receive the intervention than those who received the supplements.

Community-based supplementary feeding programs in the resource poor settings have been proven to be effective in improving the height, weight and height-for-age Z score score in a period of 6 months in the previous reported studies^(14)^. Consistent with this, we found a statistically significant increase in height, i.e. 8·5 cm and LAZ score, i.e. 0·19 over a period of 6 months. A systematic review on the effectiveness of supplementary food also reported an increase in height-for-age Z score, i.e. 0·15 in disadvantaged children below 2 years of age^(15)^. A study conducted in urban slums of Haiti reported a mean increase of 0·13 in LAZ score over a period of 6 months, while the children in the 3 months group did not show any effect on the LAZ score^(16)^. Another study conducted in Wenchuan, China, reported the effectiveness of ready-to-use supplementary food (RUSF) on the prevention of stunting in children below 2 years age in an emergency context^(17)^.

A cluster randomised controlled trial conducted in Chad reported that adding RUSF as part of general food distribution will result in improvement of linear growth. In this study, the baseline characteristics showed increased height-for-age Z score in the intervention arm −1·65 (1·56 sd) compared with the control arm −1·85 (1·43 sd)^(18)^, while there was no difference in the weight and height of the participants, consistent with our findings.

A community-based randomised control trial conducted in Sindh, Pakistan, reported a 9 % reduction in the stunting and relative risk of 0·83 (95 % CI 0·81, 0·86) with a locally produced supplement. Consistent with this, we found that the LNS reduced stunting by 7 % and the HR was 2·4 (95 % CI 1·66, 3·48) for participants in the control arm. The intervention in both studies was similar^(19)^. A systematic review conducted in 2019 reported that LNS along with complementary feeding reduced the risk of moderate stunting by 7 % and severe stunting by 15 % (Relative Risk (RR) 0·93 and 0·85, respectively). It has been suggested that LNS as part of general food distribution is effective in improving the growth outcomes in children below 2 years age^(20)^.

The study in Sindh, Pakistan, also reported that on sub-group analysis, the intervention was more protective in 6–12 months children (RR 0·83, 95 % CI 0·81, 0·86) in comparison to children 13–18 months (RR 0·90, 95 % CI 0·83, 0·97). In sub-group analysis, we found that the unadjusted RR was 0·46 (95 % CI 0·31, 0·69) for children 6–12 months in comparison to RR 0·34 (95 % CI 0·19, 0·61) for children 13–20 months. However, the HR revealed no significant difference for age of the child at enrollment in both model 1 and model 2. This may be due to differences in child age at enrollment along with low consumption of the LNS by the children below 1 year old due to the continued intermittent breast-feeding^(19)^. A similar finding was reported by an ethnographic study conducted in a Guatemalan town that strong commitment to breast-feeding was one of the obstacles to effectiveness of RUSF^(21)^. In our study, exclusive breast-feeding at baseline was 63·74 % (*n* 1069), and intact breast-feeding was 60·1 % (*n* 1004).

A study conducted in Tehran, Iran, reported the effectiveness of RUSF on growth indicators of children 24–59 months. This study reported that children who received RUSF had a lower prevalence of diarrhoea^(22)^. Consistently, we found that at 6 months there was a statistically significant difference between the prevalence of diarrhoea between the intervention and control group, i.e. 10·85 % and 15·34 %, respectively (*P* value 0·01).

After adjustment for the background characteristics, we found that male children were more likely to experience stunting (HR 1·3, 95 % CI: 1·10, 1·59, *P* value 0·003). Similarly, a study conducted in Cambodia reported that female children had statistically significant increased anthropometric values including height-for-age Z score in comparison to the participants in the control arm. Although the intervention was locally produced fish-based RUSF, the overall impact was not significant – as reported there was a high loss to follow-up in the study which could have introduced bias in the study^(23)^.

Similar studies on the efficacy of food-based interventions for the prevention of chronic malnutrition have been conducted in the developing world, but they are often carried out in controlled conditions/situations, and the generalisability of these results cannot measure the impact of such interventions under field operations. Our study was conducted as a community-based trial, with 6 months and 12 months follow-up with a good sample size providing sufficient evidence to develop policies and programmes aimed to prevent stunting in children. We hope that the evidence that we have provided will help in improving maternal and child health and growth outcomes in other countries. The RUSF was locally produced and can be easily accessible, affordable and acceptable to the community addressing the alarming situation of stunting in Pakistan.

Regarding limitations, we accept that the study was underpowered in relation to our original power calculation. However, recruitment was undertaken under challenging conditions in relation to the geographical and political context of the area in which the study was undertaken and the COVID-19 pandemic through 2020. We nevertheless feel that we have provided sufficient evidence here to support the conclusions that we have made. Another limitation of our study is that randomised control trials are the gold standard of the community-based trial, but we could not perform the randomisation due to the already allocated areas. It would have been best if random allocation was performed at the community level. Dietary data of the study participants regarding routine dietary intake were not collected regularly, which if collected would have further strengthened the results.

### Conclusion and further directions

We have here provided evidence that locally produced ready-to-use supplementary food is an effective intervention for preventing stunting in children below 2 years of age. This could be provided as part of the malnutrition prevention package to overcome stunting in Pakistan and elsewhere in the world. The RUSF can be locally produced, can be easily accessible, affordable and acceptable to the community. Due to the low cost of this ready-to-use supplementary food, this intervention could help the developing world in reducing the already alarming rates of chronic malnutrition with ongoing evaluation of anthropometric and health outcomes through childhood and beyond.

## Supporting information

Fazid et al. supplementary material Fazid et al. supplementary material

## Supplementary material

For supplementary material/s referred to in this article, please visit https://doi.org/10.1017/S0007114523002702

## References

[1] Govender I , Rangiah S , Kaswa R , et al. (2021) Malnutrition in children under the age of 5 years in a primary health care setting. S Afr Fam Pract 63, 5337. 10.4102/safp.v63i1.5337 PMC8517826 34677078

[2] Walson JL & Berkley JA (2018) The impact of malnutrition on childhood infections. Curr Opin Infect Dis 31, 231. 29570495 10.1097/QCO.0000000000000448 PMC6037284

[3] Black RE , Victora CG , Walker SP , et al. (2013) Maternal and child undernutrition and overweight in low-income and middle-income countries. Lancet 382, 427–451. 23746772 10.1016/S0140-6736(13)60937-X

[4] Sayre RK , Devercelli AE , Neuman MJ , et al. (2015) Investing in Early Childhood Development: Review of the World Bank’s Recent Experience. Washington, DC: International Bank for Reconstruction and Development/The World Bank.

[5] Hunder AA (2023) What is Acute Malnutrition?: Action Against Hunger. https://actionagainsthunger.ca/what-is-acute-malnutrition/what-is-acute-malnutrition/ (accessed January 2023).

[6] Pakistan U. National Nutrition Survey 2018 (2018) Key Findings Report. Islamabad: UNICEF Pakistan.

[7] Frongillo EA Jr , de Onis M & Hanson KM (1997) Socioeconomic and demographic factors are associated with worldwide patterns of stunting and wasting of children. J Nutr 127, 2302–2309. 9405578 10.1093/jn/127.12.2302

[8] De Onis M & Branca F (2016) Childhood stunting: a global perspective. Matern Child Nutr 12, 12–26. 27187907 10.1111/mcn.12231 PMC5084763

[9] ISRCTN (2017) Ready to Use Supplementary Foods (RUSF) to Prevent Stunting among Children under Five Years in Kurram Agency (Internet). ISRCTN Registry. https://www.isrctn.com/ISRCTN94319790 (accessed December 2022).

[10] Program WF (2018) WFP Specialized Nutritious Foods Sheet: World Food Program. https://www.kowr.gov.pl/uploads/pliki/promocja_zagraniczna/wydarzenia_inne/wfp_specialized_nutritious_foods_sheet.pdf (accessed December 2022).

[11] Khan A , Ul-Haq Z , Fazid S , et al. (2023) Effectiveness of locally produced ready to use supplementary food on hemoglobin, anthropometrics, plasma micronutrients concentrations of 6 to 23 months age children: a non-randomized community-based trial from Pakistan. Frontiers in Nutrition 10. (2296–861X (Print)). 10.3389/fnut.2023.1176778 PMC10415027 37575332

[12] Khan A , Ul-Haq Z , Fatima S , et al. (2023) Long-term impact of multiple micronutrient supplementation on micronutrient status, hemoglobin level, and growth in children 24 to 59 months of age: a non-randomized community-based trial from Pakistan. Nutrients 15, 1690. 37049531 10.3390/nu15071690 PMC10096793

[13] University of Aberdeen Research Tools 2017 (Projects undertaken by the unit have contributed to the development of many different research tools for both researchers and users of research. in collaboration with colleagues at other universities we have developed tools to help trialists match their trial design decisions to the needs of the intended users of the trial results.). https://www.abdn.ac.uk/hsru/what-we-do/tools/index.php#panel177 (accessed October 2017).

[14] Keats EC , Das JK , Salam RA , et al. (2021) Effective interventions to address maternal and child malnutrition: an update of the evidence. Lancet Child Adolesc Health 5, 367–384. 33691083 10.1016/S2352-4642(20)30274-1

[15] Visser J , McLachlan MH , Maayan N , et al. (2018) Community-based supplementary feeding for food insecure, vulnerable and malnourished populations–an overview of systematic reviews. The Cochrane Database of Systematic Reviews, issue 11, CD010578. 30480324 10.1002/14651858.CD010578.pub2 PMC6517209

[16] Iannotti LL , Dulience SJL , Green J , et al. (2014) Linear growth increased in young children in an urban slum of Haiti: a randomized controlled trial of a lipid-based nutrient supplement. Am J Clin Nutr 99, 198–208. 24225356 10.3945/ajcn.113.063883 PMC3862455

[17] Dong C , Ge P , Ren X , et al. (2013) Prospective study on the effectiveness of complementary food supplements on improving status of elder infants and young children in the areas affected by Wenchuan earthquake. PLoS One 8, e72711. 24039797 10.1371/journal.pone.0072711 PMC3767697

[18] Huybregts L , Houngbé F , Salpéteur C , et al. (2012) The effect of adding ready-to-use supplementary food to a general food distribution on child nutritional status and morbidity: a cluster-randomized controlled trial. PLoS Med 9, e1001313. 23028263 10.1371/journal.pmed.1001313 PMC3445445

[19] Khan GN , Kureishy S , Ariff S , et al. (2020) Effect of lipid-based nutrient supplement—Medium quantity on reduction of stunting in children 6–23 months of age in Sindh, Pakistan: a cluster randomized controlled trial. PLoS One 15, e0237210. 32790725 10.1371/journal.pone.0237210 PMC7425934

[20] Das JK , Salam RA , Hadi YB , et al. (2019) Preventive lipid-based nutrient supplements given with complementary foods to infants and young children 6 to 23 months of age for health, nutrition, and developmental outcomes. The Cochrane Database of Systematic Reviews, issue 5, CD012611. 31046132 10.1002/14651858.CD012611.pub3 PMC6497129

[21] Davis T , Fischer E , Rohloff P , et al. (2014) Chronic malnutrition, breastfeeding, and ready to use supplementary food in a Guatemalan Maya town. Hum Org 73, 72–81.

[22] Azimi F , Esmaillzadeh A , Alipoor E , et al. (2020) Effect of a newly developed ready-to-use supplementary food on growth indicators in children with mild to moderate malnutrition. Public Health 185, 290–297. 32712460 10.1016/j.puhe.2020.06.025

[23] Borg B , Sok D , Mihrshahi S , et al. (2020) Effectiveness of a locally produced ready-to-use supplementary food in preventing growth faltering for children under 2 years in Cambodia: a cluster randomised controlled trial. Matern Child Nutr 16, e12896. 31885221 10.1111/mcn.12896 PMC7038903

