# Supplementary material for: Effectiveness of locally produced ready-to-use supplementary foods on the prevention of stunting in children aged 6–23 months: a community-based trial from Pakistan
Source: Br J Nutr. 2023 Nov 28;131(7):1189–95. doi: 10.1017/S0007114523002702 (PMC10918516; doi:10.1017/S0007114523002702)
Supplement: Fazid et al. supplementary material [file S0007114523002702sup001.docx]

**Supplementary table 1: Composition of nutrient values in Lipid Based Nutrition Supplement (RUSF)**

| Nutrients values per 50 g product (one serving) | Unit | Minimum | Maximum |
| --- | --- | --- | --- |
| Energy | kcal | 255 | 280 |
| Protein | g | 5.5 | 8 |
| Fat | g | 13 | 18 |
| ω-3 fatty acids | g | 0.15 | 0.9 |
| ω-6 fatty acid | g | 1.3 | 3.1 |
| Retinol (VitaminA) | mcg | 275 | 575 |
| Thiamin (VitaminB1) | mg | 0.5 | - |
| Riboflavin (Vitamin B2) | mg | 1.05 | - |
| Niacin (VitaminB3) | mg | 6.5 | - |
| Pantothenic Acid (Vitamin B5) | mg | 2 | - |
| Pyridoxine (Vitamin B6) | mg | 0.9 | - |
| Biotin (VitaminB7) | mcg | 30 | - |
| Folate (VitaminB9) | mcg | 165 | - |
| Cobalamin (Vitamin B12) | mcg | 1 | - |
| Ascorbate (VitaminC) | mg | 30 | - |
| Cholecalciferol (Vitamin D) | mcg | 7.5 | 10 |
| Tocopherol Acetate (Vitamin E) | mg aTE | 8 | - |
| Phytomenadione (Vitamin K) | mcg | 13.5 | - |
| Calcium (Ca) | mg | 268 | 375 |
| Copper (Cu) | mg | 0.7 | 1.0 |
| Iodine (I) | mcg | 50 | 70 |
| Iron (Fe) | mg | 5 | 7 |
| Magnesium (Mg) | mg | 75 | 113 |
| Manganese (Mn) | mg | 0.6 | 1.2 |
| Phosphorus (P) | mg | 225 | 375 |
| Potassium (K) | mg | 450 | 700 |
| Selenium (Se) | mcg | 10 | 20 |
| Sodium (Na) | mg | - | 135 |
| Zinc (Zn) | mg | 5.5 | 7 |
